# Ustekinumab trough levels in children with Crohn’s disease refractory to anti-tumor necrosis factor agents: a prospective case series of off-label use

**DOI:** 10.3389/fphar.2023.1180750

**Published:** 2023-09-25

**Authors:** Marleen Bouhuys, Paola Mian, Patrick F. van Rheenen

**Affiliations:** ^1^ Department of Pediatric Gastroenterology, Hepatology and Nutrition, Beatrix Children’s Hospital, University of Groningen, University Medical Centre Groningen, Groningen, Netherlands; ^2^ Department of Clinical Pharmacy and Pharmacology, University Medical Centre Groningen, Groningen, Netherlands

**Keywords:** ustekinumab, therapeutic drug monitoring, Crohn’s disease, inflammatory bowel disease, pediatrics

## Abstract

**Background:** Ustekinumab is used off-label in pediatric Crohn’s disease refractory to anti-tumor necrosis factor. Data on optimal dosing, target trough levels, and potential benefit of therapeutic drug monitoring in children treated with ustekinumab are limited.

**Materials and Methods:** We describe a series of six adolescents who consented to be treated with ustekinumab. We measured their trough levels, C-reactive protein, and fecal calprotectin before every administration.

**Results:** Standard adult dosing was effective to achieve biochemical remission (fecal calprotectin < 250 mg/kg) in one patient and clinical remission (resolution of symptoms) in another. The other four patients failed to respond on standard dosing and underwent intravenous re-induction and interval shortening to increase ustekinumab trough levels. This resulted in biochemical remission in one patient and clinical remission in another, suggesting an exposure–response relationship. The remaining two patients had no therapeutic benefit, and ustekinumab was discontinued.

**Conclusion:** In this report, we show that ustekinumab can induce remission in pediatric patients with anti-tumor necrosis factor refractory Crohn’s disease. It is worth escalating the dose before abandoning the drug as ineffective. Prospective studies in children are needed to determine long-term efficacy of ustekinumab, usefulness of therapeutic drug monitoring strategies, and, if applicable, optimal target trough levels.

## 1 Introduction

The incidence of pediatric Crohn’s disease (CD) has increased rapidly over the past decades (Kuenzig et al., 2022). During this same period, anti-tumor necrosis factor (TNF) has become a treatment option which has positively altered the natural disease course of CD. While the cumulative five-year exposure rate to anti-TNF has increased to more than 50%, fewer patients now develop stricturing complications or require ileal resections (L et al., 2022). Despite these recent advances, therapeutic challenges arise in children refractory or intolerant to anti-TNF therapy.

According to the European guideline on the medical management of pediatric CD, ustekinumab can be considered in patients who fail to achieve or maintain clinical remission on adequately dosed anti-TNF agents (infliximab or adalimumab) in combination with immunomodulator use (van Rheenen et al., 2020). Ustekinumab is a fully human monoclonal antibody that targets the p40 protein subunit of interleukin-12 and -23 (European Medicines Agency, 2022). In a systematic review of 63 real-world studies in mostly adult refractory CD patients, ustekinumab was found to be safe and effective. Response was achieved in 64% and remission in 45% after ∼1 year (Rubín de Célix et al., 2022). Ustekinumab is not authorized for the treatment of CD in children (European Medicines Agency, 2022). It is, therefore, prescribed off-label, and data on optimal dosing, target drug levels, and attainment of adequate trough levels in children are limited (Carman et al., 2018).

Therapeutic drug monitoring (TDM) of monoclonal antibodies is based on observations that higher trough drug concentrations are associated with higher efficacy and that loss of response is primarily attributed to either low drug levels or to the development of anti-drug antibodies (i.e., immunogenicity). TDM has been proven to increase the efficacy and decrease the toxicity of anti-TNF therapy (Kapoor and Crowley, 2021). Studies on adults suggest that ustekinumab may be suitable for TDM, based on an extensive inter- and intraindividual variability in pharmacokinetics and the presence of an exposure–response relationship (Vande Casteele et al., 2017; Adedokun et al., 2018; Gómez Espín et al., 2021; Alsoud et al., 2022; Liefferinckx et al., 2022; Proietti et al., 2022). Development of anti-ustekinumab antibodies is uncommon (Bots et al., 2021).

There is paucity of information on the relationship between drug exposure levels and response in children. We here report on our experiences with off-label use of ustekinumab in six pediatric CD patients refractory to anti-TNF.

## 2 Materials and methods

This is a prospective case series in which patient care and data collection were planned ahead of time. In accordance with the European guideline, children with CD who were refractory to anti-TNF therapy were treated with a single intravenous dose of ustekinumab (6 mg/kg rounded to 130 mg, maximum 520 mg), followed by a subcutaneous injection of 90 mg every 8 weeks at our out-patient department (van Rheenen et al., 2020). 30 min before each ustekinumab administration, ustekinumab trough concentration and C-reactive protein (CRP) were measured. Ustekinumab trough concentrations were measured in serum with the use of an enzyme-linked immunosorbent assay (ELISA) by Sanquin Diagnostic Services (Amsterdam, Netherlands), and the detection range was 0.005–20 μg/mL (Menting et al., 2015). Fecal calprotectin was measured every 2–3 months or when a patient developed symptoms of a flare. Baseline characteristics, including disease and treatment history, fecal calprotectin, and CRP before ustekinumab initiation, were extracted from the patients’ medical records. Clinical remission was defined as a complete resolution of symptoms. Biochemical remission was defined as a fecal calprotectin below 250 mg/kg (van Rheenen et al., 2020). Patients who turned 18 continued their treatment at the adult gastroenterology department where routine trough level measurements were not performed.

This project concerned the evaluation of existing healthcare. When participating in a healthcare evaluation, patients were exposed to neither additional research procedures nor additional risks. Written informed consent was obtained for the off-label use of ustekinumab and for the publication of any potentially identifiable data included in this article.

## 3 Results

A total of six patients, five of which were male, were initiated on ustekinumab between November 2019 and June 2022 at a median age of 15.5 years (range 11–17). The median fecal calprotectin at ustekinumab initiation was 2072 mg/kg (range 1314–6000). Median follow-up after ustekinumab initiation was 29 months (range 4–35). None of the patients underwent endoscopy during follow-up on ustekinumab. No side effects of ustekinumab were observed.

**FIGURE 1:**
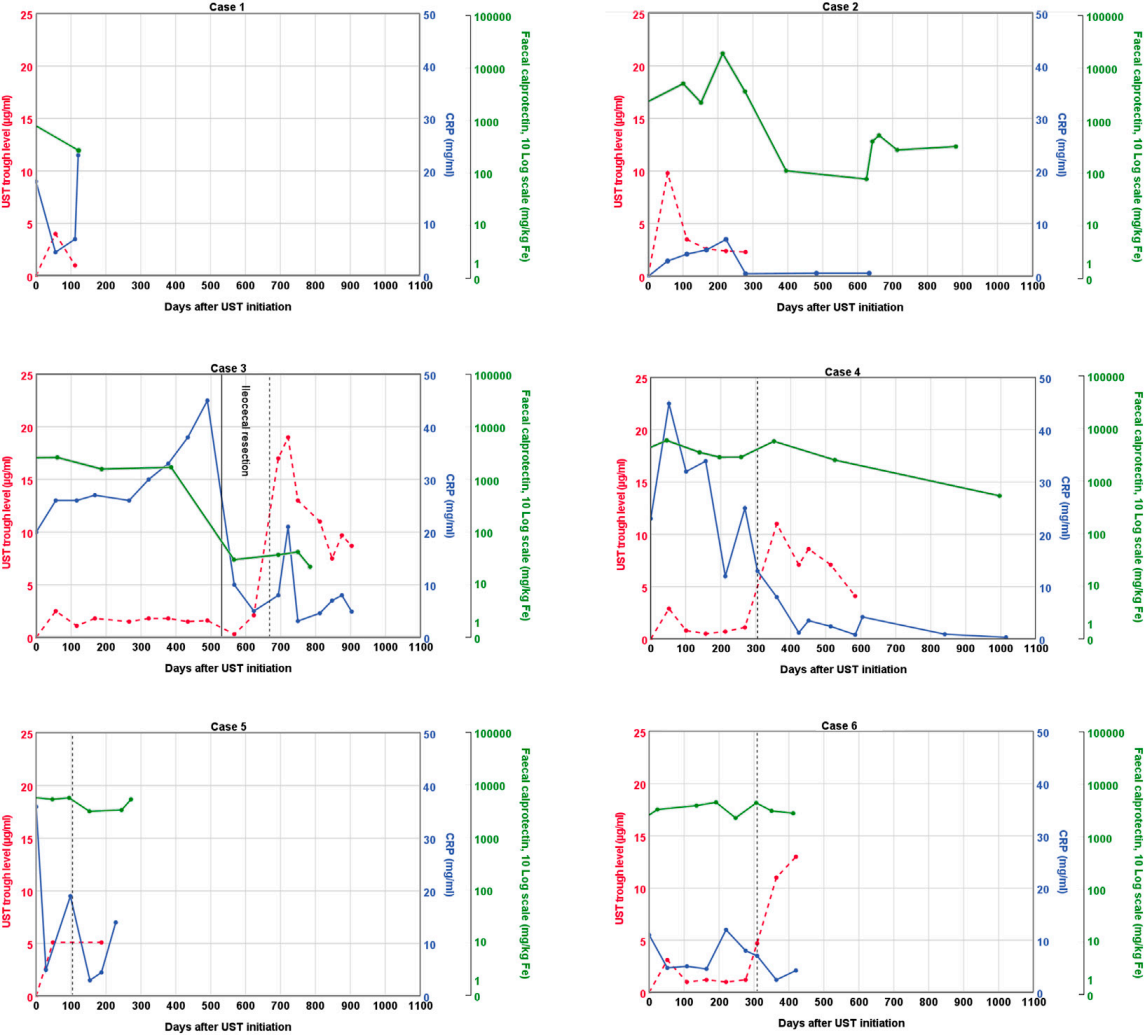
Graphical representation of ustekinumab trough levels, fecal calprotectin, and C-reactive protein over time. The vertical dashed line represents a second intravenous loading dose of ustekinumab followed by a shortened subcutaneous ustekinumab interval of 4 weeks. UST: ustekinumab; CRP; C-reactive protein.

A summary of the clinical information before and at initiation of ustekinumab is presented in Table 1 and Table 2. Figure 1 shows ustekinumab trough levels, fecal calprotectin, and CRP plotted over time. An overview of all ustekinumab administrations, dosing, and measurements is provided in the Supplementary Material. A detailed description of the cases is provided in the following.

**TABLE 1:** Clinical information before initiation of ustekinumab.

Case	Sex	Age at diagnosis (in years)	Presenting symptoms	Paris classification at diagnosis (Levine et al., 2011)	Fecal calprotectin at diagnosis (in mg/kg)	Induction treatment	Maintenance treatment
1	Male	12	Abdominal pain; diarrhea	L3B2G0	3925	EEN	Infliximab + azathioprine
						Prednisone	Infliximab
						Infliximab	Adalimumab
2	Male	13	Diarrhea; rectal bleeding; abdominal pain	L3B1G0	450	Prednisone	Infliximab + azathioprine
						EEN	Adalimumab
						Infliximab	
3	Male	11	Perianal fistula and abscess; anemia	L3+4aB1pG0	795	Infliximab	Infliximab + azathioprine
							Adalimumab + azathioprine
4	Male	11	Abdominal pain; constipation; decreased appetite; weight loss	L1+4bB1G0	3600	EEN	Infliximab + azathioprine
						Prednisone	Adalimumab + azathioprine
						Infliximab	
5	Female	13	Weight loss; diarrhea; aphthous stomatitis	L2B1pG1	?	Infliximab	Infliximab + azathioprine
							Vedolizumab
6	Male	9	Erythema nodosum; arthritis; diarrhea; fever	L3B0G0	7460	Prednisone	Infliximab + azathioprine
						Infliximab	

EEN: exclusive enteral nutrition.

**TABLE 2:** Clinical information at ustekinumab initiation.

Case	Age (in years)	Height (in cm)	Weight (in kg)	Fecal calprotectin (in mg/kg)	C-reactive protein (in mg/mL)	MINI index	Co-medication	Time since diagnosis (in months)
1	17	189	80	1530	4.5	18	-	64
2	16	168	62	1390	<0.3	16	-	51
3	14	159	44	2613	20	14	Azathioprine	31
4	16	171	77	6220	23	14	-	52
5	15	173	67	>6000	36	?	-	26
6	11	153	42	1314	11	18	Azathioprine	16

MINI: mucosal inflammation noninvasive index (Cozijnsen et al., 2020).

### 3.1 Case 1

Case 1 presented at age 12 with abdominal pain and diarrhea. At endoscopy, inflammation of the terminal ileum and the right-sided colon was seen. Initial induction treatment included EEN and azathioprine maintenance (2 mg/kg). Because of lack of response to EEN, a corticosteroid-tapering scheme was followed. Despite an initial clinical improvement, symptoms quickly reappeared after steroid withdrawal. A third induction treatment, this time with infliximab (5 mg/kg at week 0, 2, and 6 followed by every 8 weeks) was started and azathioprine was continued. After the third infusion, a symptomatic stenosis of the terminal ileum was found, which necessitated ileocecal resection. Infliximab monotherapy was given postoperatively to reduce the risk of recurrent Crohn’s inflammation of the anastomosis. Azathioprine was discontinued after shared decision making. Two years later, low titer anti-infliximab antibodies were detected. Infliximab treatment escalation made the antibodies disappear. Unfortunately, after another year, an anaphylactic reaction occurred during infliximab administration and treatment was switched to adalimumab (80 mg, followed by 40 mg every 2 weeks). Because of persistently subtherapeutic trough levels (2.4–3 μg/mL), the adalimumab dose was increased (80 mg every 2 weeks). This resulted in adequate trough levels (7.5 μg/mL) and biochemical remission. One year after the initiation of adalimumab, the patient developed immunogenic loss of response due to the formation of anti-adalimumab antibodies. Ileocolonoscopy revealed active disease in the neo-terminal ileum and ascending colon. Adalimumab was discontinued, and ustekinumab was initiated. An intravenous loading dose of 390 mg was administered, followed by 90 mg subcutaneously every 8 weeks. Currently, 5 months after the initiation of ustekinumab, the patient is in clinical and biochemical remission, with an ustekinumab trough level of 1 μg/mL.

### 3.2 Case 2

Case 2 presented at age 12 with diarrhea, rectal bleeding, and abdominal pain. At endoscopy, Crohn’s colitis and inflammation of the ileocecal valve were seen. Initial induction treatment included a corticosteroid-tapering regimen and azathioprine (2 mg/kg). After an initial clinical improvement, rectal bleeding reappeared as soon as steroids were withdrawn. A second induction treatment, this time with exclusive enteral nutrition (EEN), was also unsuccessful, and a step-up to infliximab was made (5 mg/kg at week 0, 2 and 6, followed by maintenance every 8 weeks). The patient reached biochemical remission before the fourth infusion and maintained remission until 1.5 years later. Despite dose and interval escalation (to 10 mg/kg every 6 weeks, with a trough level of 4.9 μg/m), rectal bleeding occurred and fecal calprotectin was >5000 mg/kg. Infliximab was switched to adalimumab (80 mg followed by 40 mg every 2 weeks), azathioprine was stopped, and remission was re-achieved. Eighteen months later, loss of response was observed. Dose escalation (to 80 mg) and interval shortening (to weekly dosing) did not yield any clinical benefit anymore. An intravenous loading dose of ustekinumab of 390 mg was administered, followed by 90 mg subcutaneously every 8 weeks. Ustekinumab trough levels in the maintenance phase were stable around 2.5 μg/mL. Symptoms of distal inflammation (feeling of urgency and worries of incontinence) improved after a concurrent course of local budesonide. Clinical remission was reached 5 months after the initiation of ustekinumab, but fecal calprotectin levels remain mildly elevated.

### 3.3 Case 3

Case 3 presented at age 11 with perianal disease (fistula and abscess) and anemia. At endoscopy, terminal ileitis and aphthous lesions of the stomach and duodenum were seen in addition to perianal disease. He was treated with upfront high-dose infliximab (10 mg/kg at week 0, 2, and 6), followed by infliximab maintenance (5 mg/kg) every 8 weeks and azathioprine (2 mg/kg). Clinical remission was achieved, but fecal calprotectin, erythrocyte sedimentation rate, and CRP remained elevated. After 1.5 years of infliximab and azathioprine combination therapy, both luminal and perianal disease recurred. A within-class switch to adalimumab was to no avail, despite high adalimumab trough levels, and azathioprine co-medication continued. Fecal calprotectin was 2613 mg/kg when ustekinumab was introduced. An intravenous loading dose of 260 mg was administered, followed by 90 mg subcutaneously every 8 weeks. Almost 1.5 years after ustekinumab was initiated, the terminal ileum was still severely inflamed and stenotic, and a new perianal abscess had formed. Ustekinumab trough levels at that time were between 1.5 and 1.8 μg/mL. Ileocecal resection with primary ileocolic anastomosis was complicated by an anastomotic leak, which resulted in a temporary diverting ileostomy. Ustekinumab and methotrexate were continued after surgery. Four months later, a small bowel obstruction was observed, consistent with active inflammation in the loop in front of ileostomy. A second intravenous ustekinumab loading dose was administered, followed by subcutaneous ustekinumab (90 mg) every 4 weeks. Ustekinumab trough levels increased dramatically (between 7.5 and 19 μg/mL). Currently, fecal calprotectin is normal, perianal fistulae have closed, and the ileostomy has successfully been reversed.

### 3.4 Case 4

Case 4 presented at age 11 with abdominal pain, constipation, decreased appetite, weight loss, and increased fecal calprotectin. At endoscopy, Crohn’s inflammation was seen in the stomach and terminal ileum. Initial induction treatment included EEN and azathioprine maintenance (2.5 mg/kg), but remission was not achieved. A second induction treatment with a corticosteroid-tapering scheme was also unsuccessful. A third induction treatment, this time with infliximab (5 mg/kg at week 0, 2, and 6 followed by every 8 weeks) resulted in complete resolution of symptoms, but fecal calprotectin levels remained high. Ileocolonoscopy confirmed persistent active inflammation in the terminal ileum. Infliximab trough levels were low (<1 μg/mL) and remained low despite dose increase (to 10 mg/kg). A switch to adalimumab did not change the situation. Terminal ileitis progressed to a significant stenosis, which necessitated an ileocecal resection. Postoperative treatment consisted of adalimumab (80 mg every 2 weeks) and azathioprine. Eight months after surgery, mild stenotic symptoms reoccurred. Adalimumab and azathioprine were discontinued, and an ustekinumab intravenous loading dose of 390 mg was administered, followed by 90 mg subcutaneously every 8 weeks. Trough levels varied between 0.5 and 1.1 μg/mL in the maintenance phase. Ten months after the first administration of ustekinumab, clinical remission was achieved, but fecal calprotectin levels remained high. After a second intravenous loading dose of ustekinumab, followed by subcutaneous ustekinumab (90 mg) every 4 weeks, ustekinumab trough levels were between 4.1 and 8.6 μg/mL. Currently, fecal calprotectin levels still remain high, but the patient refuses further colonoscopic evaluation and treatment adjustments because of the absence of symptoms.

### 3.5 Case 5

Case 5 presented at age 13 with weight loss, diarrhea, and aphthous stomatitis. At colonoscopy, colonic inflammation and perianal fistulae were seen. Because of perianal involvement, a starting dose of 10 mg/kg infliximab was administered. At week 2 and 6, a dose of 5 mg/kg was used, followed by a maintenance dose of 5 mg/kg every 8 weeks. Because of primary non-response, empiric dose intensification and interval shortening were employed, but remission was not achieved. Infliximab and azathioprine co-medication were discontinued, and vedolizumab was initiated. Multiple hospitalizations were required to manage disease flares with corticosteroids or exclusive enteral nutrition. High fecal calprotectin levels persisted, and endoscopically confirmed panenteric ulcerations necessitated a switch to ustekinumab. An intravenous loading dose of 390 mg was administered, followed by 90 mg subcutaneously every 8 weeks. Sixteen weeks after the loading dose, neither clinical nor biochemical response was observed. The ustekinumab trough level at that time was 5.1 μg/ml. A second intravenous ustekinumab loading dose was administered, followed by 90 mg subcutaneously every 4 weeks. Despite treatment intensification, ustekinumab trough levels did not increase, fecal calprotectin remained high, and perianal disease persisted. Ustekinumab was discontinued because of primary non-response, and adalimumab (160 mg followed by 80 mg every 2 weeks) and azathioprine (1.5 mg/kg) were initiated. Currently, 4 months after the initiation of adalimumab, remission has still not been achieved.

### 3.6 Case 6

Case 6 presented at age 9 with erythema nodosum, arthritis of the ankle, diarrhea, and fever. At endoscopy, extensive inflammation primarily affecting the terminal ileum and cecum was seen. Initial induction treatment included a corticosteroid tapering regimen and azathioprine (2.3 mg/kg). After an initial clinical improvement, symptoms reoccurred during steroid tapering. A second induction treatment, this time with infliximab (5 mg/kg at week 0, 2, and 6 followed by every 8 weeks), was started. Remission was not achieved despite optimizing infliximab trough levels. Infliximab was discontinued, and ustekinumab was initiated. An intravenous loading dose of 260 mg was administered, followed by 90 mg subcutaneously every 8 weeks. Azathioprine was continued. After five subcutaneous administrations of ustekinumab and trough levels between 1.0 and 1.5 μg/mL, a second intravenous loading dose was administered, followed by subcutaneous injections every 4 weeks. This resulted in a dramatic rise in trough levels (11–13 μg/mL). Nevertheless, colonic inflammation persisted which resulted in an endoscopically impassable stenosis of the ascending colon. Ustekinumab was discontinued, and ileocolic resection was performed. Postoperative treatment consisted of adalimumab (80 mg, followed by 40 mg every 2 weeks) and azathioprine (2 mg/kg) to reduce the risk of postoperative recurrence. Moreover, all fecal calprotectin levels were in the target range (<250 mg/kg).

## 4 Discussion

We presented six pediatric cases with luminal CD who were refractory to at least one anti-TNF agent. Loss of response to anti-TNF occurs commonly. Four out of the six cases (Carman et al., 2018; van Rheenen et al., 2020; European Medicines Agency, 2022; Kuenzig et al., 2022) developed stricturing complications at a relative young age and required bowel resection. Case 3 and 5 had additional perianal involvement. All six cases represent a CD clinical phenotype with a less favorable prognosis. In patients with this CD phenotype, the traditional step-wise or “pyramidal” approach almost inevitably leads to the top of the pyramid with no remaining medication options unless an off-label drug, such as ustekinumab, is used. We acknowledge that the anti-TNF treatment in the two patients with perianal involvement may have been suboptimal. Higher postinduction infliximab trough levels have been associated with better response in perianal fistulizing disease (El-Matary et al., 2019). Although these two patients did receive an initial starting dose of 10 mg/kg infliximab, this higher dosing was de-escalated to standard dosing after only 1 or 3 infusions.

Ustekinumab therapy using standard adult dosing was effective to achieve biochemical remission in case 1 and clinical remission in case 2. Intravenous re-induction and interval shortening of ustekinumab in the other four patients resulted in biochemical remission in case 3 and clinical remission in case 4. Case 5 and 6 had no therapeutic benefit of ustekinumab, despite dose escalation, and ustekinumab was discontinued.

### 4.1 Effectiveness of ustekinumab in pediatric Crohn’s disease

As previously summarized, two out of the six patients achieved biochemical remission on ustekinumab, another two achieved clinical remission, and in the remaining two, ustekinumab was discontinued for lack of response. In the latter two, case 5 and 6, treatment was withdrawn after 9 and 15 months, respectively, which was sufficiently long to observe a therapeutic response. According to the updated therapeutic targets in IBD, a mean of 11–14 weeks after ustekinumab initiation is required to achieve the medium-term treatment target clinical remission and normalization of fecal calprotectin (to <250 mg/kg) and C-reactive protein (Turner et al., 2021). Endoscopic remission, which was not measured in our patients, represents an even deeper form of remission and is considered a long-term treatment outcome which can be achieved after 19 weeks (Turner et al., 2021).

In a retrospective cohort study, effectiveness of ustekinumab was evaluated in 42 CD patients aged 21 years or below (median 16.9, interquartile range 13.9–18.2). 74% patients were still on ustekinumab after 1 year. 60% achieved steroid-free remission, and 67% achieved clinical remission (Dayan et al., 2019). Another cohort study in children showed similar results (Kim et al., 2021). In a third cohort study, only 39% of pediatric CD patients achieved clinical remission 1 year after ustekinumab initiation (Chavannes et al., 2019). Meta-analyses of studies among adult patients with active perianal CD suggested that ustekinumab may also be beneficial in this category of patients (Attauabi et al., 2021; Godoy Brewer et al., 2021). Prospective clinical trials are needed to confirm the long-term efficacy of ustekinumab in the treatment of refractory pediatric CD.

### 4.2 Treatment escalation

Four out of the six children failed on ustekinumab standard dosing and consequently underwent intravenous re-induction and interval shortening. This resulted in a substantial increase in ustekinumab levels at week 4 in three cases (>10 μg/mL) and a slight revert in the maintenance phase, but with a net increase compared to the standard dosing regimen. To the best of our knowledge, this is the first study in children in which ustekinumab drug levels were routinely measured. Higher drug levels resulted in two additional patients with a favorable response, which suggested an exposure–response relationship. In case 5, dose escalation had no effect on trough levels.

Our escalation strategy consisted of intravenous re-induction, followed by a shortened interval of subcutaneous ustekinumab of 4 weeks. This strategy was also described in a retrospective case study in which seven out of 10 children treated with ustekinumab required escalation, although the dosing interval was only shortened to 5 or 6 weeks (Do et al., 2020). In a retrospective cohort of 69 children that failed on ustekinumab standard dosing, escalation also mainly consisted of interval shortening (91%). A combination of intravenous re-induction and interval shortening was performed on only three patients. 42% of the pediatric cohort achieved clinical remission within 3 months after escalation. Adverse events were rare, mild, and did not result in ustekinumab discontinuation (Yerushalmy-Feler et al., 2022).

### 4.3 Target trough levels

Establishing an exposure–response relationship is the first step in developing a TDM strategy. Multiple studies have demonstrated such a relationship of ustekinumab in adult CD patients (Battat et al., 2017; Adedokun et al., 2018; Verstockt et al., 2019; Painchart et al., 2020; Thomann et al., 2020; Gómez Espín et al., 2021; Yao et al., 2021; Hirayama et al., 2022; Liefferinckx et al., 2022), and our report additionally could support an exposure–response relationship in children. Next, target trough levels must be determined. Unfortunately, those target levels for ustekinumab in children have not been described.

With the aim to expand our knowledge of ustekinumab (target) levels in children, we measured trough levels before each administration. Trough levels 8 weeks after the first intravenous loading dose (i.e., before the first subcutaneous administration) varied in our patients, ranging from 2.5 to 9.8 μg/mL (mean 4.6 μg/mL). Trough levels during maintenance (i.e., at 16 weeks or later) with standard ustekinumab dosing ranged from 0.3 to 4.7 μg/mL.

In the absence of specific target trough levels for children, adult targets were often used. For ustekinumab, a variety of target trough levels have been appointed in adults, partly depending on the timing of measurement and the outcome of interest (Battat et al., 2017; Adedokun et al., 2018; Verstockt et al., 2019; Painchart et al., 2020; Thomann et al., 2020; Gómez Espín et al., 2021; Yao et al., 2021; Hirayama et al., 2022; Liefferinckx et al., 2022). An overview of these target ustekinumab trough levels and therapeutic outcomes in adult patients with CD is presented in table 3.

**TABLE 3:** Overview of ustekinumab concentration targets and therapeutic outcomes in adult patients with CD.

Study	Design	No. of patients	Timing TDM	Target trough level (in µg/mL)	Therapeutic outcome
Adedokun et al. (2018)	Post-hoc analysis of three phase 3 RCTs	1369	Maintenance (≥week 24)	>0.8–1.4	Clinical remission during maintenance
Battat et al. (2017)	Prospective and cross-sectional cohort study	62	Maintenance (≥week 26)	>4.5	CRP and endoscopic improvement
Gómez Brewer et al. (2021)	Cross-sectional cohort study	58	Maintenance (≥week 24)	>1.4	Clinical remission
				>2.0	Biochemical remission
				>2.2	Composite clinical/biochemical remission
Hirayama et al. (2022)	Retrospective cohort study	28	Maintenance	>1.7	Normal CRP and albumin levels
				>2.0	Endoscopic remission
Liefferinckx et al. (2022)	Retrospective cohort study	99	Week 16	>1.2	Clinical outcome (treatment status)
				>1.6	Endoscopic outcome
Painchart et al. (2020)	Prospective cohort study	49	Week 12 (induction: SC 0, 4, 12)	≥1.1	Biological response within 6 months
Thomann et al. (2020)	Retrospective cohort study	72	Week 8	≥2	Clinical response at week 16
Verstockt et al. (2019)	Prospective cohort study	86	Week 8	≥4.2	50% decrease in fecal calprotectin
			Week 16	≥2.3	Endoscopic response after 6 months
			Week 24	≥1.9	Endoscopic response after 6 months
Yao et al., 2021	Retrospective cohort study	19	Week 16/20	>1.12	Endoscopic remission

TDM: therapeutic drug monitoring.

If ustekinumab trough level measurements are performed before the first subcutaneous injection (i.e., 8 weeks after intravenous induction), trough levels ≥2 μg/mL are associated with clinical response at week 16 and ≥4.2 with a 50% decrease in fecal calprotectin (Verstockt et al., 2019; Thomann et al., 2020). Ustekinumab levels of our patients at this time point ranged from 2.5 to 9.8 μg/mL.

During maintenance (i.e., ≥16 weeks after induction), target trough levels >0.8–1.4 μg/mL are associated with clinical remission (Adedokun et al., 2018; Gómez Espín et al., 2021). Ustekinumab trough levels ≥4.5 are associated with biochemical response (Battat et al., 2017), trough levels >1.7–2.0 with biochemical remission, (Gómez Espín et al., 2021; Hirayama et al., 2022), >1.6–4.5 with endoscopic response (Battat et al., 2017; Verstockt et al., 2019; Liefferinckx et al., 2022), and trough levels >1.12–2.0 with endoscopic remission (Yao et al., 2021; Hirayama et al., 2022). This is consistent with the trough levels found in case 2 of our series, who is in clinical remission on standard dosing. Case 1 from our series, who is in clinical and biochemical remission on standard dosing, has a slightly lower trough level of 1 μg/mL.

### 4.4 Timing of trough level measurement

Overall, two strategies for TDM are distinguished: proactive and reactive TDM. In proactive TDM, drug serum concentrations are routinely measured, irrespective of disease activity. Proactive TDM early in the treatment course allows for early dose optimization which can result in increased efficacy. Reactive TDM is performed if a disease flare occurs to help determine the cause of the flare and the next therapeutic step (such as increasing the dose or shortening the interval, or switching to an out-of-class biological) (van Rheenen et al., 2020; Shmais et al., 2022). Looking at the recent history of infliximab, the development of reactive TDM strategies will most likely precede that of proactive TDM strategies (Shmais et al., 2022). It has to be noted that within our six cases, we measured ustekinumab trough levels routinely before each subcutaneous injection, but the decision to escalate the dose was primarily based on clinical and biochemical parameters. Trough levels were measured without adapting dosing based on trough levels alone, which did not fully comply with the TDM criteria. Ustekinumab trough levels, however, were considered when deciding to discontinue ustekinumab.

Based on the currently available data, clear recommendations for the timing of trough level measurement of ustekinumab in pediatric CD cannot be given. Although substantiated by limited evidence, some recommendations for adults have been made. In a study by Cheifetz *et al.* a panel of 10 adult gastroenterology experts reached consensus that reactive TDM should be performed in patients with confirmed primary non-response to ustekinumab (prior to switching therapy) and in patients with confirmed secondary loss of response, but the panel did not formulate a target level or corresponding therapeutic step (Cheifetz et al., 2021). Shukla *et al.* proposed to proactively monitor ustekinumab levels, but the level of evidence to support this approach was low (Shukla and Ananthakrishnan, 2021).

Because of the potential exposure–response relationship in children, ustekinumab may be suitable for TDM in children. The findings in this report, together with contradicting results in adults, endorse the need for more data about ustekinumab trough levels in children. As an important first step, clinical centers that have the means should routinely measure ustekinumab drug levels in all children treated with ustekinumab and collect their findings in (multicenter) registries or databases.

### 4.5 Immunomodulator co-medication

In two of our patients, an immunomodulator was continued during ustekinumab treatment. The most important indication for an immunomodulator during treatment with a biological response is to prevent the formation of anti-drug antibodies. However, as mentioned before, immunogenicity in ustekinumab is less common than in anti-TNF therapy (Yarur et al., 2022). None of our patients developed anti-ustekinumab antibodies. Based on the currently available literature, combination treatment with an immunomodulator does not seem to have added value compared to ustekinumab monotherapy (Rosh et al., 2021; Yarur et al., 2022).

### 4.6 Limitations of this study

Shortcomings of case series, including a small sample size and lack of a control group, also apply to this case series. This limits generalizability and precludes drawing firm conclusions. Moreover, in a limited number of patients, follow-up time was relatively short for some patients.

Although the methods of ustekinumab prescription and monitoring of effectiveness were recorded in the local protocol, we had to rely on data accumulated in the electronic patient file. Since such records often are not complete, important data may be missing, which introduces a source of error into the report.

The intention of this report was to describe and discuss ustekinumab trough level measurement, not to prove that TDM is the way to go. Observations conducted in the six cases reported here cannot be generalized to other patients.

### 4.7 Conclusions

In this report, we show that ustekinumab can induce remission in pediatric CD patients refractory to anti-TNF therapy. Treatment escalation can induce remission in patients non-responsive to the standard treatment, supporting an exposure–response relationship. Treatment escalation should, therefore, be considered before the drug is abandoned as ineffective. Prospective clinical trials in children are needed to determine the long-term efficacy of ustekinumab, optimal target drug concentrations, and TDM strategies.

## Data Availability

The original contributions presented in the study are included in the article/Supplementary Material; further inquiries can be directed to the corresponding author.
