# Supplementary material for: Ustekinumab trough levels in children with Crohn’s disease refractory to anti-tumor necrosis factor agents: a prospective case series of off-label use
Source: Front Pharmacol. 2023 Sep 25;14:1180750. doi: 10.3389/fphar.2023.1180750 (PMC10561290; doi:10.3389/fphar.2023.1180750)
Supplement: Supplementary file 1 [file Table1.DOCX]

Supplementary Material

**Ustekinumab Trough Levels in Children with Crohn’s Disease Refractory to Anti-Tumor Necrosis Factor Agents: a Prospective Case Series of Off-Label Use**

**Marleen Bouhuys, Paola Mian, Patrick F. van Rheenen^*^**

*** Correspondence:** Patrick F. van Rheenen: p.f.van.rheenen@umcg.nl

# Supplementary Data

| Patient | Days since UST initiation | Intravenous dose UST (in mg) | Subcutaneous dose UST (in mg) | UST concentration (in µg/ml) | Fecal calprotectin (in mg/kg) | C-reactive protein (in mg/ml) | Erythrocyte sedimentation rate (in mm/h) |
| --- | --- | --- | --- | --- | --- | --- | --- |
| 1 | -88 | - | - | - | 1530 | - |  |
|  | 0 | 390 | - | 0.0 | - | 18.0 | 17 |
|  | 56 | - | 90 | 4.0 | - | 4.5 | 7 |
|  | 112 | - | 90 | 1.0 | - | 7.0 | 15 |
|  | 121 | - | - | - | 240 | 23.0 |  |
| 2 | -51 | - | - | - | 1390 | - |  |
|  | 0 | 390 | - | 0.0 | - | 0.0 |  |
|  | 56 | - | 90 | 9.8 | - | 2.9 | 13 |
|  | 99 | - | - | - | 4466 | - |  |
|  | 150 | - | - | - | 1952 | - |  |
|  | 111 | - | 90 | 3.5 | - | 4.2 | 16 |
|  | 167 | - | 90 | 2.6 | - | 5.0 | 15 |
|  | 212 | - | - | - | 16680 | - |  |
|  | 223 | - | 90 | 2.4 | - | 7.0 | 15 |
|  | 276 | - | - | - | 3180 |  |  |
|  | 279 | - | 90 | 2.3 | - | 0.5 | 4 |
|  | 335 | - | 90 | - | - | - |  |
|  | 391 | - | 90 | - | - | - |  |
|  | 394 | - | - | - | 98 | - |  |
|  | 447 | - | 90 | - | - | - |  |
|  | 482 | - | - | - | - | 0.6 | 3 |
|  | 503 | - | 90 | - | - | - |  |
|  | 559 | - | 90 | - | - | - |  |
|  | 615 | - | 90 | - | - | - |  |
|  | 624 | - | - | - | 68 | - |  |
|  | 633 | - | - | - | - | 0.6 | 2 |
|  | 641 | - | - | - | 355 | - |  |
|  | 660 | - | - | - | 465 | - |  |
|  | 671 | - | 90 | - | - | - |  |
|  | 712 | - | - | - | 245 | - |  |
|  | 727 | - | 90 | - | - | - |  |
|  | 783 | - | 90 | - | - | - |  |
|  | 839 | - | 90 | - | - | - |  |
|  | 880 | - | - | - | 285 | - |  |
|  | 895 | - | 90 | - | - | - |  |
|  | 951 | - | 90 | - | - | - |  |
| 3 | -134 | - | - | - | 2530 | - |  |
|  | 0 | 260 | - | 0.0 | - | 20.0 | 35 |
|  | 56 | - | 90 | 2.5 | - | 26.0 | 40 |
|  | 60 | - | - | - | 2613 |  |  |
|  | 116 | - | 90 | 1.1 | - | 26.0 | 40 |
|  | 117 | - | 90 | - | - |  |  |
|  | 168 | - | 90 | 1.8 | - | 27.0 | 27 |
|  | 187 | - | - | - | 1570 |  |  |
|  | 266 | - | 90 | 1.5 | - | 26.0 | 28 |
|  | 322 | - | 90 | 1.8 | - | 30.0 | 26 |
|  | 378 | - | 90 | 1.8 | - | 33.0 | 17 |
|  | 386 | - | - | - | 1700 |  |  |
|  | 434 | - | 90 | 1.5 | - | 38.0 | 24 |
|  | 490 | - | 90 | 1.6 | - | 45.0 | 25 |
|  | 566 | - | - | - | 29 |  |  |
|  | 567 | - | 90 | 0.3 | - | 10.0 | 41 |
|  | 623 | - | 90 | 2.1 | - | 5.0 | 11 |
|  | 668 | 260 | - | - | - |  |  |
|  | 693 | - | 90 | 17.0 | 36 | 8.0 | 7 |
|  | 721 | - | 90 | 19.0 |  | 21.0 | 7 |
|  | 749 | - | 90 | 13.0 | 41 | 3.1 | 8 |
|  | 784 | - | 90 | - | 21 | - |  |
|  | 812 | - | 90 | 11.0 | - | 4.6 | 7 |
|  | 847 | - | 90 | 7.5 | - | 7.0 | 11 |
|  | 875 | - | 90 | 9.7 | - | 8.0 | 13 |
|  | 903 | - | 90 | 8.7 | - | 4.9 | 9 |
| 4 | -41 | - | - | - | 3490 | - |  |
|  | 0 | 390 | - | 0.0 | - | 23 | 27 |
|  | 46 | - | - | - | 6220 | - |  |
|  | 52 | - | 90 | 2.9 | - | 45 | 42 |
|  | 101 | - | 90 | 0.8 | - | 32 | 42 |
|  | 140 | - | - | - | 3678 |  |  |
|  | 157 | - | 90 | 0.5 | - | 34 | 46 |
|  | 197 | - | - | - | 2969 | - |  |
|  | 213 | - | 90 | 0.7 | - | 12 | 35 |
|  | 259 | - | - | - | 2985 | - |  |
|  | 269 | - | 90 | 1.1 | - | 25 | 43 |
|  | 305 | 390 | - | - | - | 13 | 31 |
|  | 332 | - | 90 | - |  | - |  |
|  | 353 | - | - | - | 5990 | - |  |
|  | 360 | - | 90 | 11 | - | 8 | 28 |
|  | 395 | - | 90 | - | - | - |  |
|  | 423 | - | 90 | 7.1 | - | 1.2 | 12 |
|  | 451 | - | 90 | 8.6 | - | 3.5 | 13 |
|  | 486 | - | 90 | - | - | - |  |
|  | 514 | - | 90 | 7.1 | - | 2.4 | 12 |
|  | 527 | - | - | - | 2620 | - |  |
|  | 542 | - | 90 | - | - | - |  |
|  | 584 | - | 90 | 4.1 | - | 0.8 | 9 |
|  | 605 | - | - | - | - | 4.2 | 15 |
|  | 612 | - | 90 | - | - | - |  |
|  | 640 | - | 90 | - | - | - |  |
|  | 668 | - | 90 | - | - | - |  |
|  | 696 | - | 90 | - | - | - |  |
|  | 731 | - | 90 | - | - | - |  |
|  | 759 | - | 90 | - | - | - |  |
|  | 787 | - | 90 | - | - | - |  |
|  | 815 | - | 90 | - | - | - |  |
|  | 840 | - | - | - | - | 0.9 | 3 |
|  | 843 | - | 90 | - | - | - |  |
|  | 871 | - | 90 | - | - | - |  |
|  | 899 | - | 90 | - | - | - |  |
|  | 927 | - | 90 | - | - | - |  |
|  | 955 | - | 90 | - | - | - |  |
|  | 983 | - | 90 | - | - | - |  |
|  | 998 | - | - | - | 540 | - |  |
|  | 1011 | - | 90 | - | - | - |  |
|  | 1015 | - | - | - | - | 0.3 | 3 |
| 5 | -23 | - | - | - | 6000 |  |  |
|  | 0 | 390 | - | 0.0 | - | 36.0 | 31 |
|  | 28 | - | - | - | - | 5.0 | 17 |
|  | 47 | - | - | - | 5420 | - |  |
|  | 48 | - | 90 | 5.1 | - | - |  |
|  | 95 | - | - | - | 5775 | - |  |
|  | 98 | - | - | - | - | 19.0 | 30 |
|  | 104 | 390 | - | - | - | - |  |
|  | 132 | - | 90 | - | - | - |  |
|  | 153 | - | - | - | 3204 | - |  |
|  | 154 | - | - | - | - | 3.0 | 10 |
|  | 160 | - | 90 | - | - | - |  |
|  | 187 | - | 90 | 5.1 | - | 4.5 | 18 |
|  | 215 | - | 90 | - | - | - |  |
|  | 228 | - | - | - | - | 14.0 | 31 |
|  | 243 | - | 90 | - | - | - |  |
|  | 245 | - | - | - | 3390 | - |  |
|  | 272 | - | 90 | - | 5410 | - |  |
| 6 | -61 | - | - | - | 1314 | - |  |
|  | 0 | 260 | - | 0.0 | - | 11 | 26 |
|  | 24 | - | - | - | 3166 | - |  |
|  | 52 | - | 90 | 3.1 | - | 4.7 | 20 |
|  | 108 | - | 90 | 1.0 | - | 5 | 18 |
|  | 136 | - | - | - | 3760 | - |  |
|  | 164 | - | 90 | 1.2 | - | 4.5 | 19 |
|  | 192 | - | - | - | 4350 | - |  |
|  | 220 | - | 90 | 1.0 |  | 12 | 23 |
|  | 248 | - | - | - | 2180 | - |  |
|  | 276 | - | 90 | 1.2 |  | 8 | 21 |
|  | 307 | - | - | - | 4240 | - |  |
|  | 309 | 260 | - | 4.7 | - | 7 | 18 |
|  | 336 | - | 90 | - | - | - |  |
|  | 351 | - | - | - | 2980 | - |  |
|  | 364 | - | 90 | 11.0 | - | 2.4 | 10 |
|  | 392 | - | 90 | - | - | - |  |
|  | 413 | - | - | - | 2680 | - |  |
|  | 420 | - | 90 | 13.0 | - | 4.2 | 9 |
|  | 448 | - | 90 | - | - | - |  |
